# Glycolipids slow interfacial proton migration while preserving surface proton retention

**DOI:** 10.1073/pnas.2537390123

**Published:** 2026-07-08

**Authors:** Anna Maznichenko, Peter Pohl

**Affiliations:** ^a^Institute of Biophysics, Department of Physics, Johannes Kepler University Linz, Linz 4040, Austria

**Keywords:** confined water, membrane transport, bioenergetics, fluorimetry, diffusion

## Abstract

Proton gradients power diverse biological processes, yet how interfacial proton migration is regulated remains unclear. Here we quantify how membrane composition controls interfacial proton migration using an approach that releases protons directly at the surface of a membrane patch via an embedded ionophore. Fluorometrically monitoring proton arrival at a distant patch across neutral, negatively charged, and positively charged membranes, we confirm that both the lateral surface diffusion coefficient and the activation barrier for proton release into the bulk vary rather modestly. In contrast to membrane electrostatics, membrane incorporation of glycolipids typical of thylakoid membranes—digalactosyldiacylglycerol and sulfoquinovosyldiacylglycerol—leads to a more pronounced reduction of the lateral proton diffusion coefficient, with comparatively small effects on the surface-to-bulk release barrier. Thus, interfacial proton migration is governed primarily by hydration-layer properties rather than membrane charge. These results establish membrane-anchored sugars as potent modulators of long-range proton conduction and provide a mechanistic framework for localized proton coupling in glycolipid-rich biological membranes.

## Significance

Protons can migrate along membrane surfaces to link distant proton pumps and proton-consuming enzymes, but the membrane features that control this migration are not well understood. While membrane charge exerts only a limited influence, we now demonstrate that membrane-anchored sugars markedly slow interfacial proton migration without compromising proton retention at the interface. These findings identify hydration-layer organization as a key control knob for proton conduction and suggest a physical basis by which sugar-rich membranes can spatially tune proton-powered bioenergetics.

Proton gradients energize a wide range of essential biological processes by storing electrochemical free energy that can be converted into directed transport or mechanical work. The mechanistic basis of proton-coupled transport has been elucidated in detail for several systems. In bacterial sugar/H^+^ symporters such as lactose permease, structural and functional studies established an alternating-access mechanism in which proton binding is tightly coupled to substrate translocation (1). Similarly, proton-coupled oligopeptide transporters such as PepT1 exploit H^+^ gradients for intestinal nutrient uptake (2). The bacterial translocon SecYEG has likewise been proposed to harness a transmembrane proton gradient to drive polypeptide transport (3). In plants, sucrose/H^+^ symporters load photoassimilates into the phloem, linking extracellular pH and membrane potential to long-distance carbohydrate distribution (4). Proton gradients also power ATP synthesis, as F-type and related V-type rotary ATPases convert proton flow into mechanical rotation that drives ATP formation (5, 6).

In bacteria, mitochondria, and chloroplasts, proton gradients are generated by membrane-embedded proton pumps. Coupling between proton sources and sinks can occur either through diffusion in the aqueous bulk or via lateral proton migration along membrane surfaces (7, 8). Energetically, surface migration appears favorable and has been invoked to explain several longstanding puzzles in bioenergetics (9). For example, alkalophilic bacteria sustain high ATP synthesis rates despite a proton motive force seemingly too small to support chemiosmotic coupling (10). In mitochondria, mild uncoupling can markedly reduce transmembrane pH gradients—thereby limiting reactive oxygen species—without compromising ATP production, consistent with localized proton transfer near the cristae membrane (11). Direct visualization of lateral pH gradients between proton pumps and ATP synthase further supports localized interfacial proton migration (12). Near proton sources and sinks, however, equilibrium is often not reached, and proton transport is governed by nonequilibrium dynamics. Under such conditions, protons can be transiently confined to the membrane interface, promoting lateral migration prior to exchange with the bulk (13).

Such confined proton fluxes cannot be explained by transport models based on diffusing protonated carriers, such as buffer molecules (14). Because diffusion of such carriers is slow, these models predict surface diffusion coefficients that are orders of magnitude too small (15). In contrast, direct experimental studies have demonstrated interfacial proton diffusion coefficients *D*_2_ on the order of ~5 × 10^–5^ cm^2^ s^–1^ (14, 16, 17), raising the question of how protons achieve mobilities approaching those in pure water. It is now widely accepted that interfacial proton migration proceeds via a Grotthuss-type hopping mechanism within the membrane hydration layer (14, 18).

Although the interfacial preference of protons has long been recognized (13), the structural origin of the Gibbs activation free-energy barrier Δ*G*^‡^ that opposes the exchange of interfacial protons with the bulk aqueous phase remains incompletely understood. Early models attributed Δ*G*^‡^ to titratable interfacial groups (19). This view was revised when experiments showed that such groups are not required in lipid bilayers (16) and that even lipid-free interfaces, such as decane–water, exhibit similarly slow proton exchange with the bulk (20).

Temperature-dependent measurements revealed that Δ*G*^‡^ is dominated by entropic contributions (21). The molecular origin of this entropic barrier remains unclear. One proposed scenario invokes dynamic restructuring of the hydrogen-bond network within the membrane hydration layer (22, 23). At equilibrium, proton accumulation at interfaces is governed by the free-energy difference ΔG between bulk and interfacial states. Many aqueous interfaces, including air–water, oil–water, and other hydrophobic interfaces, exhibit a negative surface potential, indicating preferential stabilization of hydroxide over hydronium ions (18). However, measurements using spatially resolved pH probes show no steady-state proton enrichment beyond Gouy–Chapman predictions under equilibrium conditions (24).

Near proton sources and sinks, however, equilibrium may not be reached, and proton transport is governed by nonequilibrium dynamics arising from kinetic asymmetry. Transient proton enrichment has been observed during the propagation of proton pulses near membranes (25–27), indicating that Δ*G*^‡^ delays exchange relative to lateral migration. This kinetic asymmetry—fast lateral transport versus slow release into the bulk—leads to transient confinement of protons at the interface.

At the molecular level, proton transport is inherently coupled to dynamic restructuring of hydrogen-bond networks. Rather than requiring preformed pathways, excess protons transiently generate their own conduction pathways within interfacial water (28). Simulations further show that proton mobility arises from a dynamic ensemble of states, in which strongly associated protons are effectively immobilized, while a more weakly bound population supports rapid lateral transport (29). This behavior is consistent with bulk proton transport, where proton motion is coupled to concerted hydrogen-bond rearrangements (30).

**Fig. 1.**
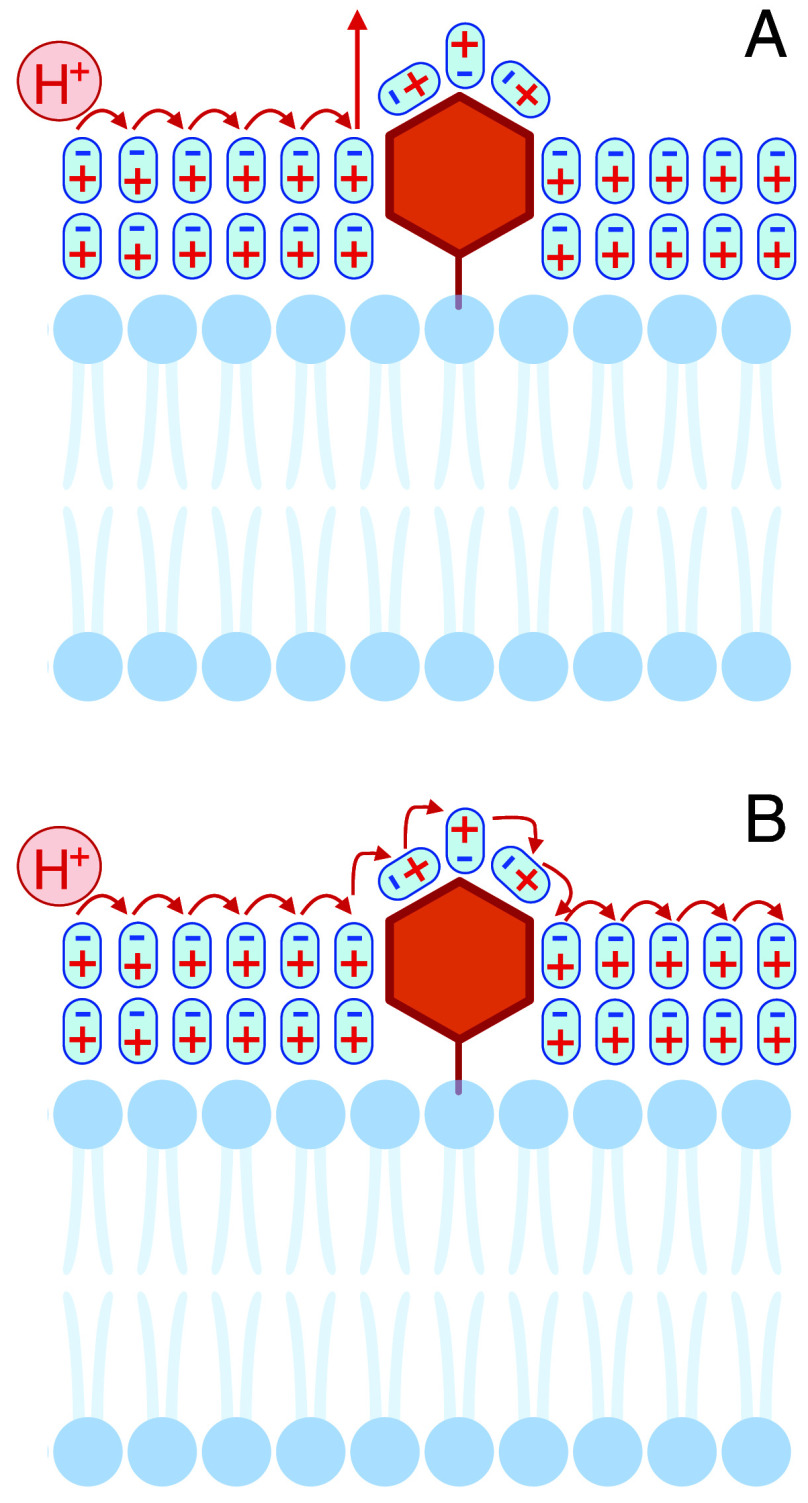
Two possible scenarios for membrane proton migration in the presence of interfacial sugar moieties. (*A*) Sugar moieties perturb the local organization of interfacial water, potentially leading to the release of protons from phospholipid-associated hydration shells and enhanced exchange with the bulk. (*B*) Alternatively, disruption of a continuous hydration layer may promote stronger interactions between protons and sugar-associated hydroxyl groups, resulting in reduced interfacial mobility. Figure created in BioRender. Pohl, P. (2026) https://BioRender.com/dolc95i.

Sugars are of particular interest in this context, as they are abundant lipid-anchored components of biological membranes. Experimental and computational studies show that sugars can strongly perturb hydration structure by forming long-lived hydrogen-bond networks with water (31). At higher concentrations, sugars form dense hydration shells that slow water dynamics, with relaxation times increased by several-fold relative to bulk water (32). Incorporation of sugar headgroups into lipid bilayers is therefore expected to modify the interfacial hydration layer and influence proton transport along membrane surfaces (Fig. 1).

In phosphocholine bilayers, the interface supports a relatively continuous hydration layer organized by phosphate and carbonyl groups, enabling efficient lateral proton hopping (14, 16). In contrast, glycolipids replace parts of this hydration layer with sugar–water clusters, creating a more heterogeneous hydrogen-bond network (33). Proton conduction pathways are thus expected to become shorter and less connected, leading to reduced lateral mobility. These effects are anticipated to depend strongly on glycolipid content, ranging from modest perturbations at low concentrations to pronounced slowing in sugar-rich membranes.

Glycolipids are particularly abundant in photosynthetic membranes. Thylakoid membranes contain approximately 50% monogalactosyldiacylglycerol (MGDG) and 25% digalactosyldiacylglycerol (DGDG), whereas the remaining fraction consists of sulfoquinovosyldiacylglycerol (SQDG) and phosphatidylglycerol, whose relative proportions vary among species (34). Proton gradients across these glycolipid-rich bilayers regulate ATP synthesis as well as photoprotective mechanisms such as nonphotochemical quenching (35). Whether enrichment in interfacial sugar moieties affects localized proton coupling relevant for these processes remains an open question. Although localized proton coupling has been proposed in thylakoids (36), proton transfer is often assumed to proceed via the bulk aqueous phase. This distinction is difficult to resolve experimentally because the aqueous space between stacked thylakoid membranes is extremely narrow (~4 nm) (10), effectively limiting the presence of bulk water. Moreover, thylakoid ATP synthase activity remains robust even after membrane potential collapse, relying primarily on the pH gradient rather than the electrical potential (37, 38).

**Fig. 2.**
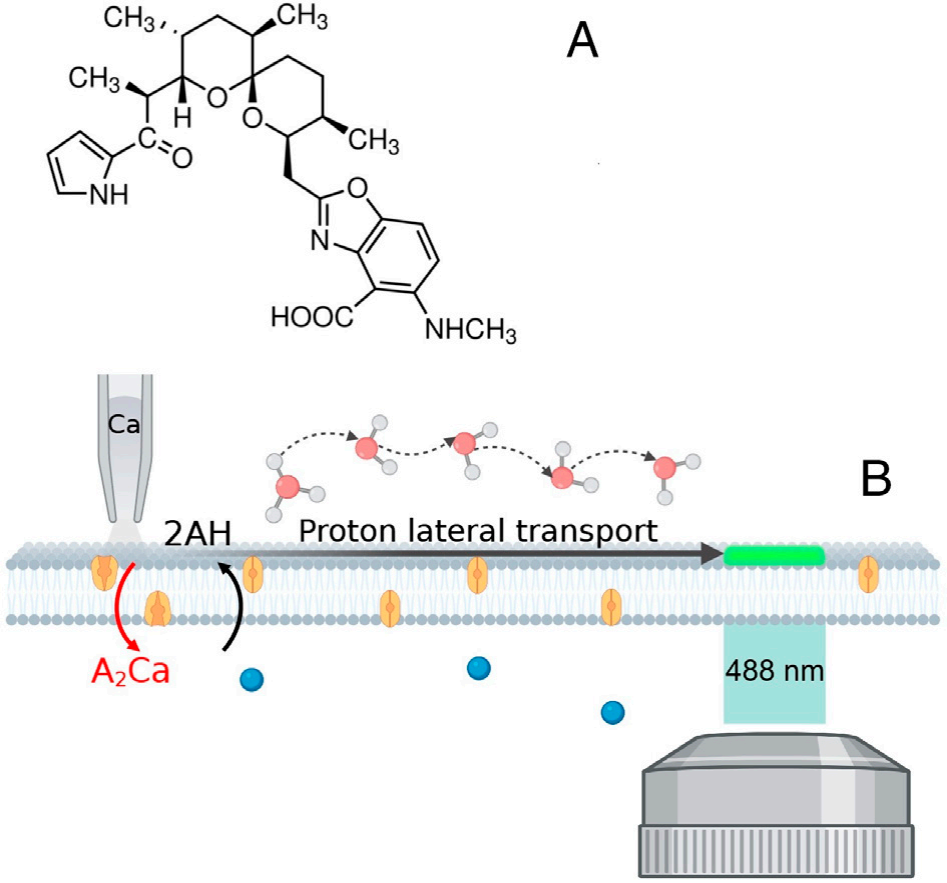
Experimental approach. (*A*) Structural formula of the calcium ionophore A23187. (*B*) Schematic of the experiment: Microinjection of calcium results in a localized increase in Ca^2+^ concentration in the upper compartment. EGTA in the aqueous phase chelates excess Ca^2+^, thereby restricting activation of A23187 (denoted *A*) to a small membrane patch directly beneath the micropipette. A23187 mediates Ca^2+^ transport toward the lower compartment and countertransport of H^+^ in the opposite direction. After dissociation from A23187, H^+^ propagates laterally along the membrane surface before entering the bulk solution. The arrival of H^+^ at a membrane patch located at a defined distance from the release site is detected as a decrease in fluorescence of pH-sensitive dyes anchored to membrane lipids.

Here, we directly examine how membrane-anchored sugars influence interfacial proton migration by quantifying both lateral proton diffusion and surface-to-bulk exchange kinetics in a system where protons are generated locally at the membrane interface. Specifically, we test whether interfacial sugar moieties promote proton release from phospholipid-associated hydration shells and enhance exchange with the bulk, or instead increase proton retention at the interface by strengthening interactions with sugar-associated hydroxyl groups and thereby slowing lateral transport (Fig. 1). To this end, we introduce an experimental approach that employs the mobile Ca^2+^/H^+^ exchanger A23187 (calcimycin) as a membrane-embedded proton source (39, 40). Unlike acid microinjection or uncaging methods, which generate protons in the bulk or near the interface, this approach produces protons directly at the membrane surface and thus more closely mimics physiological proton transfer (Fig. 2).

Using this approach, we show that glycolipid headgroups substantially reduce the lateral diffusion coefficient of interfacial protons while leaving the activation barrier for surface-to-bulk release largely unchanged. These results demonstrate that interfacial proton migration is robust yet highly sensitive to the structure and dynamics of the membrane hydration layer, and they identify membrane-anchored sugars as key modulators of long-range proton conduction along biological membranes.

## Results

### Microinjection Augments Ca^2+^ Concentration Locally Without Inducing Convection.

We first assessed the spatial extent of Ca^2+^ spreading following microinjection using the calcium-sensitive dye Fluo-3. A calibration curve relating fluorescence intensity to Ca^2+^ concentration was obtained in aqueous solution prior to the experiments. Because Fluo-3 is pH-sensitive, these measurements were performed in the absence of the ionophore A23187.

**Fig. 3.**
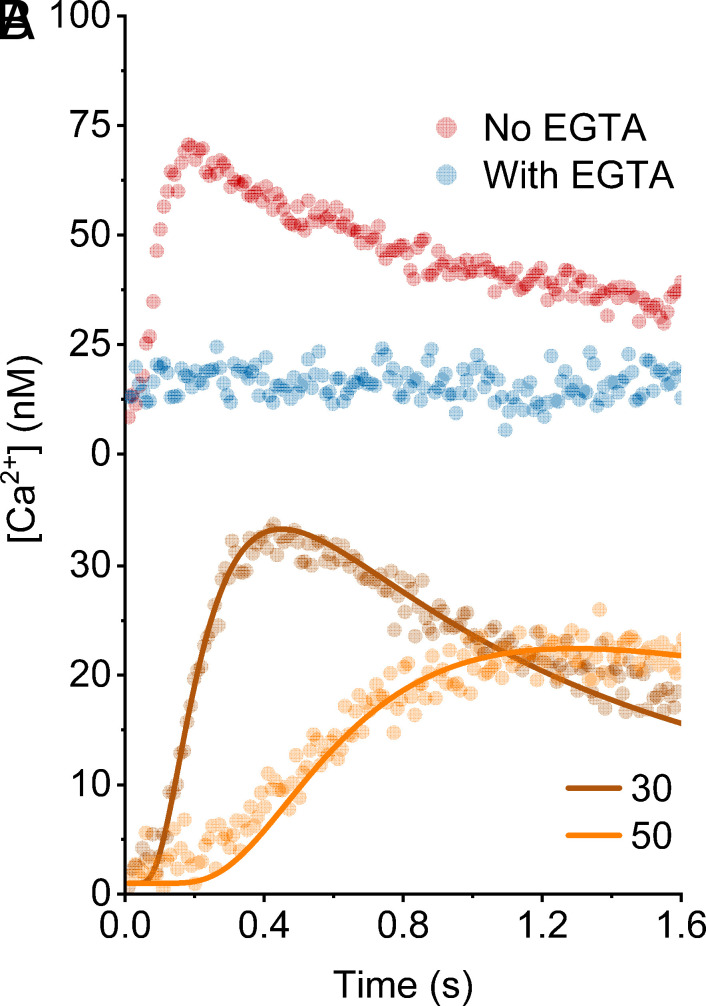
The calcium concentration (Ca^2+^) at a remote site, measured in nM, is shown as a function of time. (Ca^2+^) was calculated from fluorescence intensity changes using a previously obtained calibration curve for the Fluo-3 dye. (*A*) Comparison of experiments performed with 15 µM EGTA and without EGTA. The detection site was positioned immediately adjacent to the calcium release site. (*B*) (Ca^2+^) as a function of time measured at distances of 30 µm and 50 µm between the calcium release and detection sites. Neither EGTA nor ionophore was added. The Fluo-3 dye concentration was 5 µM. Solid lines represent fits of a three-dimensional diffusion equation (Eq. **1**), yielding a diffusion coefficient of (3.2 ± 0.1) × 10^−10^ m^2^ s^−1^.

Ca^2+^ was released from a glass micropipette positioned ~1 µm from the membrane surface. In the absence of the Ca^2+^ chelator EGTA, an increase in fluorescence was detected at a site distant from the release point (Fig. 3*A*), indicating Ca^2+^ diffusion through the aqueous phase. Addition of EGTA suppressed this spreading, confining the region of elevated Ca^2+^ concentration to a small membrane patch directly beneath the micropipette.

To verify that microinjection did not induce convection, we analyzed the kinetics of Ca^2+^ spreading from the release site to the detection site. The fluorescence time courses were downsampled (running average) to 160 data points, converted to Ca^2+^ concentrations using the calibration curve, and fitted with a three-dimensional diffusion model,

[1] $$\sigma (x,t)=\sigma_{0}+\frac{A_{\mathrm{neq}}}{(4\pi D_{3}t{)}^{\frac{3}{2}}}\exp\left(\frac{-x^{2}}{4D_{3}t}\right).$$

Here, *σ*(x,t) denotes the Ca^2+^ concentration at the detection site, *x* is the distance between the release and detection sites, and *t* is the time elapsed after microinjection. *σ_0_* is the initial Ca^2+^ concentration, *A*_neq_ is proportional to the amount of Ca^2+^ released, and *D_3_* is the Ca^2+^ diffusion coefficient. The data (Fig. 3*B*) were fitted using a modified form of (Eq. **1**) that accounts for the finite sizes of the release and detection areas, as described previously (21), yielding a diffusion coefficient *D*_3_ = 3.2 × 10^−10^ m^2^ s^−1^.

This value is approximately twofold lower than the value (7.8 × 10^–10^ m^2^s^–1^) reported for free Ca^2+^ in aqueous solution (41). The difference arises because the measured signal reflects diffusion of the Ca^2+^–Fluo-3 complex rather than free Ca^2+^. For a fast mobile buffer, the effective diffusion coefficient *D*_eff_ governing the spread of Ca^2+^ is given by (42):

[2] $$D_{\mathrm{eff}}\approx \frac{D_{\mathrm{Ca}}+\beta \;D_{B}}{1+\beta },$$

where *D*_Ca_ is the diffusion coefficient of free Ca^2+^, *D*_B_ that of Fluo-3 (or the Ca–Fluo-3 complex), and β the buffer capacity,

[3] $$\beta =\frac{B_{T}K_{d}}{{\left(K_{d}+\left[{\mathrm{Ca}}^{2+}\right]\right)}^{2}}.$$

Using a Fluo-3 concentration *B*_T_ = 5 µM and a dissociation constant *K*_d_ = 0.4 µM, we obtain β = 9. With *D*_B_ ≈ 4.9×10^−10^ m^2^ s^−1^ (43) (Eq. **2**) yields *D*_eff_ ≈ 5 ×10^−10^ m^2^ s^−1^. The remaining difference relative to the experimentally obtained value can largely be attributed to temperature differences: increasing the temperature from 19 °C (this study) to 25 °C (literature value) would increase the diffusion coefficient by ~15%, to 3.7 × 10^−10^ m^2^ s^−1^. Taken together, these results confirm that microinjection does not induce significant convection and that the experimental setup reliably resolves diffusion-limited transport.

### Ionophore-Mediated H^+^ Release from a Small Membrane Patch Confirms the Minor Role of Electrostatics in Interfacial Proton Diffusion.

We next exploited ionophore-mediated proton release to evaluate the contribution of membrane electrostatics to interfacial proton migration. Earlier studies proposed that an electrostatic barrier separates the membrane surface from the bulk aqueous phase (44), based on the observation that proton exchange rates depend on the charge of buffer molecules (45). This interpretation is difficult to reconcile with our previous findings that membrane surface charge contributes only weakly to the proton release barrier (26).

In our earlier experiments, protons were released from a hydrophobic caged compound adsorbed to the membrane surface. Differences in membrane composition could, in principle, alter probe partitioning; however, such effects are unlikely to substantially bias the relative contributions of bulk versus interfacial proton release. To eliminate even this residual ambiguity, we replaced the caged compound with the Ca^2+^/H^+^ ionophore A23187, ensuring that all protons originate from the membrane surface through Ca^2+^/H^+^ exchange across the bilayer.

**Fig. 4.**
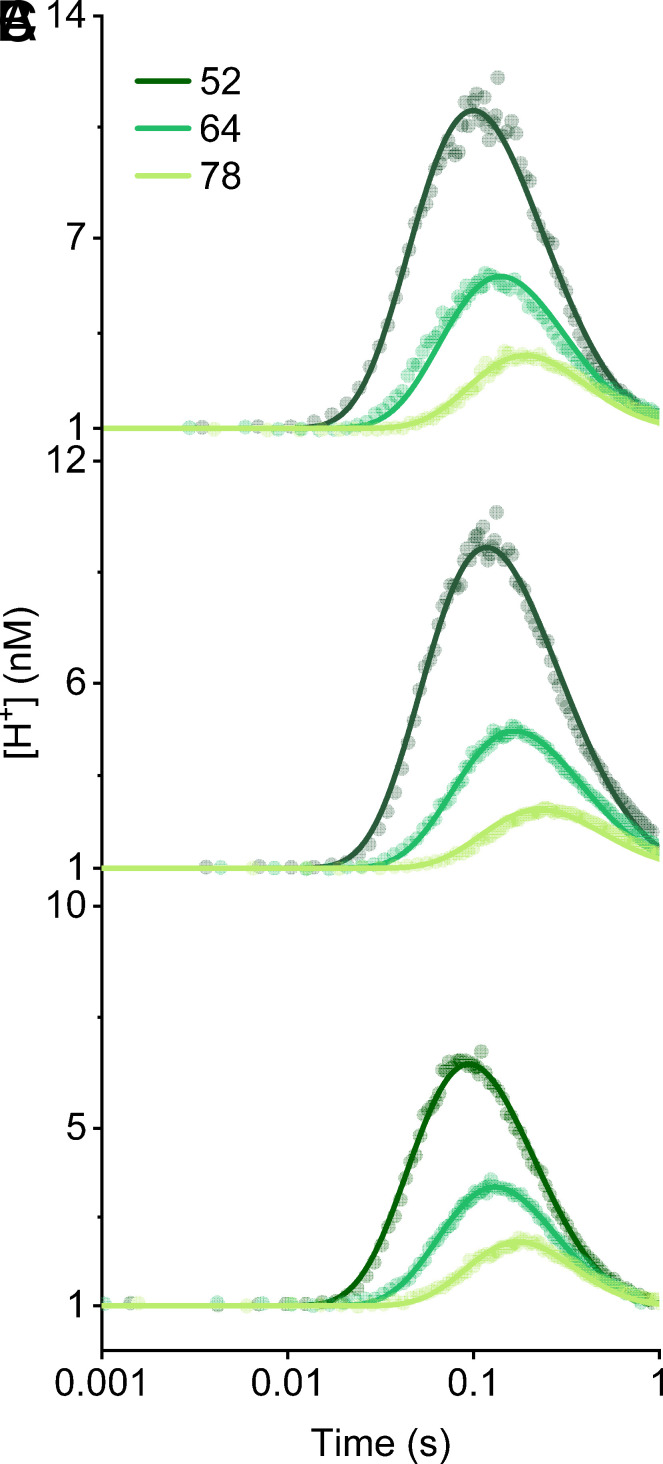
Time course of proton concentration. (H^+^), recorded at the remote detection site after Ca^2+^ microinjection at 19 °C. The three curves per panel correspond to three different distances between the detection site and the membrane patch where the ionophore was exposed to elevated (Ca^2+^) (*Inset*). Each membrane composition is shown in a separate panel: (*A*) DOPC, (*B*) DOPG, (*C*) DOTAP/DOPC in a 1:1 molar ratio. The membranes contained 3 mol% fluorescein-DHPE. Time is plotted on a logarithmic scale. Darker solid lines represent two-dimensional (2D) fits; the corresponding fit coefficients are listed in Table 1.

Experiments were performed at 19 °C on neutral (DOPC), negatively charged (DOPG), and positively charged (DOTAP/DOPC, 1:1 molar ratio) membranes containing 3% DHPE as an anchor for the pH-sensitive dye fluorescein. A 10 × 10 µm^2^ membrane patch, positioned remotely from the Ca^2+^ microinjection site, was illuminated at 488 nm (Fig. 2), and fluorescence emission was detected using a photon-counting module. Ca^2+^ microinjection initiated a propagating proton pulse (“proton wave”) at the membrane patch beneath the pipette, whose arrival at the detection site was reported by a decrease in photon count. Measurements were recorded for approximately 1 s, during which the signal returned to baseline. Each trace was averaged over eight repetitions to improve signal-to-noise ratio and plotted after applying a running exponential average (Fig. 4).

Surface proton diffusion coefficients *D*_2_ and release rate constants *k*_off_ were extracted by globally fitting the data obtained at three different source–detector distances with a two-dimensional diffusion model (25),

[4] $$\sigma \left(x,t\right)=\sigma_{0}+\frac{A_{\mathrm{neq}}}{4\pi D_{2}t}\exp\frac{-x^{2}}{4D_{2}t}\exp(-tk_{\mathrm{off}}),$$

using a modified form that accounts for the finite sizes of the release and detection areas (21). Here, *σ*(x,t) denotes the proton concentration at the detection site, *x* is the distance from the release site, and *t* is the time after microinjection. Membrane surface charge had only a modest effect on both *D*_2_ and *k*_off_ (Table 1), in agreement with previous measurements using caged protons (26). *k*_off_ was used to calculate the Δ*G*^‡^ according to transition state theory (21, 46):

[5] $$k_{\mathrm{off}}=\nu_{0}\text{exp}\left(\frac{-\Delta G^{‡}}{k_{B}T}\right).$$

**Table 1.** Parameters of lateral proton migration for different membrane compositions

Lipid	D_2_ (μm^2^ s^−1^)	*k*_off_ (s^−1^)	Δ*G*^‡^ (*kT*)
DOPC	5,338 ± 64	2.6 ± 0.1	29.0
DOPG	4,650 ± 39	1.9 ± 0.1	29.3
DOTAP 50%	5,209 ± 35	3.8 ± 0.1	28.6
SQDG 5%	3,862 ± 15	3.0 ± 0.1	28.8
SQDG 25%	3,462 ± 25	4.0 ± 0.1	28.5
DGDG 5%	3,183 ± 25	3.8 ± 0.1	28.6
DGDG 25%	1,835 ± 19	7.1 ± 0.2	28.0

Surface proton diffusion coefficients (*D*_2_), proton release rate constants (*k*_off_), and the corresponding Gibbs activation free-energy barriers (Δ*G*^‡^) for proton release from the membrane surface into the bulk extracted from two-dimensional fits to the fluorescence data. The table allows direct comparison of proton migration parameters across the different lipid compositions investigated at 19 °C. For mixed membranes, lipid fractions are given in mol%; the remaining, nonindicated fraction consists of DOPC.

where *T* is the absolute temperature, *k*_B_ is the Boltzmann constant, and *ν*_0_ ≈ 10^13^ s^−1^ is the characteristic attempt frequency for surface processes (47).

Δ*G*^‡^ varied only weakly with membrane composition, indicating that electrostatic contributions constitute only a minor fraction of the total barrier. Instead, the barrier is dominated by nonelectrostatic (primarily entropic) effects. These results confirm that electrostatics do not govern Δ*G*^‡^ and demonstrate that the ionophore-based approach yields values consistent with those obtained using caged proton release (14, 21, 26).

### Membrane-Anchored Sugar Moieties Reduce Lateral H^+^ Mobility.

**Fig. 5.**
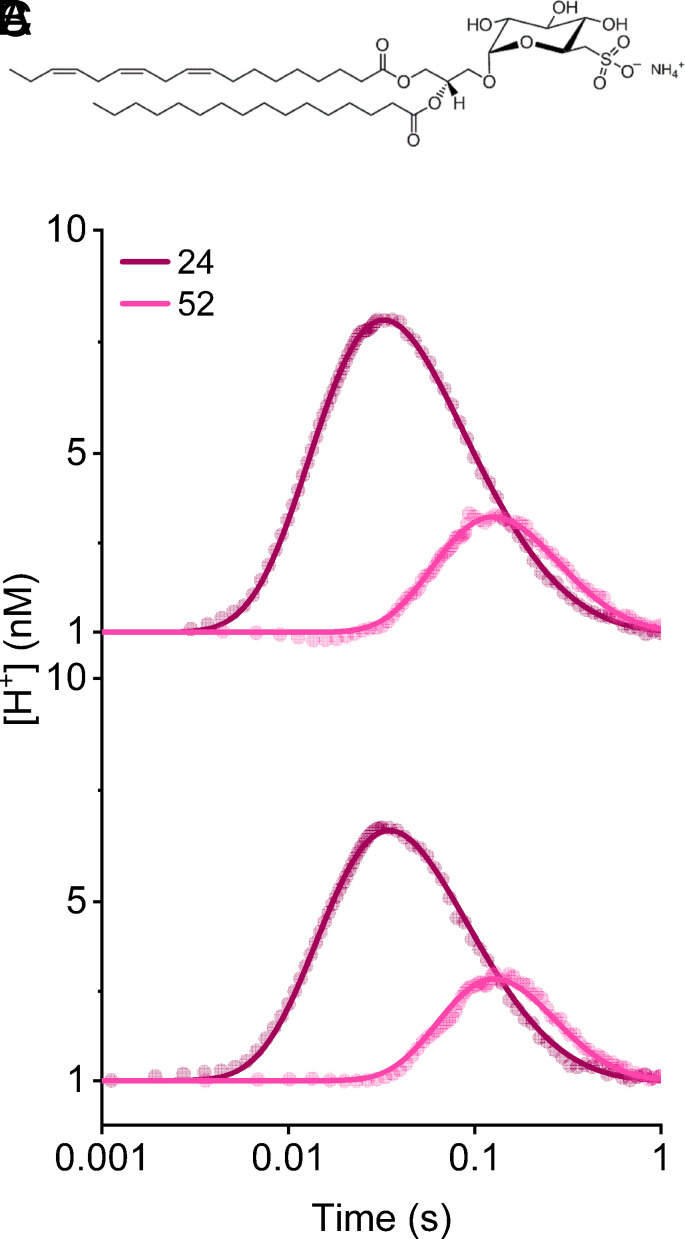
The effect of SQDG on lateral proton migration. (*A*) Chemical structure of the SQDG lipid. (*B* and *C*) Proton concentration, (H^+^), recorded at the remote detection site as a function of the time elapsed after proton release from the membrane surface at 19 °C. Each panel shows two traces corresponding to two different distances between the proton release site and the detection site; the respective distances (in µm) are indicated in the inset. Membranes contained SQDG at different mole fractions: (*B*) SQDG:DOPC (5 mol% SQDG) and (*C*) SQDG:DOPC (25 mol% SQDG). All membranes contained 3 mol% fluorescein-DHPE. Proton concentrations were calculated from fluorescence intensity changes using the corresponding calibration curves. Darker solid lines represent two-dimensional (2D) fits; the corresponding fit coefficients are listed in Table 1.

We examined the effect of membrane-anchored sugars on interfacial proton migration by incorporating the glycolipid SQDG into DOPC membranes (Fig. 5). The experimental procedure was identical to that used for Fig. 4. Compared with sugar-free membranes, the spatial extent of proton propagation was reduced, which precluded measurements at a third, more distant detection patch. The effect was moderate, with the diffusion span decreasing by less than a factor of two. Consistent with this observation, fitting the data with the two-dimensional diffusion model revealed an increased *k*_off_, corresponding—via transition state analysis—to a modest decrease in Δ*G*^‡^ (Table 1). The surface diffusion coefficient *D*_2_ also decreased, indicating reduced lateral proton mobility in the presence of sugar headgroups.

**Fig. 6.**
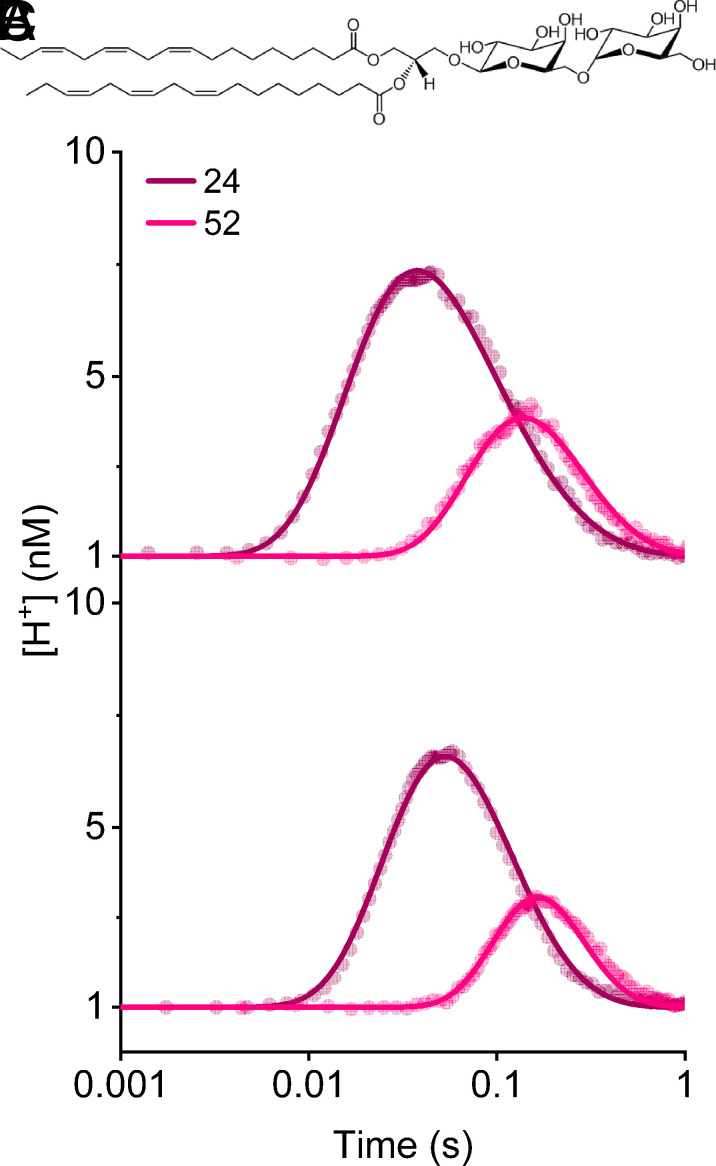
The effect of DGDG on lateral proton migration. (*A*) Chemical structure of the DGDG lipid. (*B* and *C*) Proton concentration, (H^+^), recorded at the remote detection site as a function of the time elapsed after proton release from the membrane surface at 19 °C. Each panel shows two traces corresponding to two different distances between the proton release site and the detection site; the respective distances (in µm) are indicated in the inset. Membranes contained DGDG at different mole fractions: (*B*) DGDG:DOPC (5 mol% DGDG) and (*C*) DGDG:DOPC (25 mol% DGDG). All membranes contained 3 mol% fluorescein-DHPE. Proton concentrations were calculated from fluorescence intensity changes using the corresponding calibration curves. Dark solid lines represent two-dimensional (2D) fits; the corresponding fit coefficients are listed in Table 1.

Repeating the experiments with a second glycolipid, DGDG (Fig. 6), yielded a similarly increased *k*_off_ and a small reduction in Δ*G*^‡^. However, the decrease in *D*_2_ was more pronounced (Table 1). This difference is consistent with the higher density of sugar moieties at the membrane surface, as DGDG contains two sugar headgroups per lipid whereas SQDG contains only one. The fact that the negative charge of SQDG has only a minor effect is consistent with the results obtained for charged membranes (Fig. 4). Together, these findings demonstrate that membrane-anchored sugars slow lateral proton diffusion in a concentration-dependent manner while exerting only a weak influence on the surface-to-bulk proton release barrier.

To further assess the thermodynamic contributions to proton transport, temperature-dependent measurements were performed and analyzed using Arrhenius relations. The resulting analysis, presented in the *SI Appendix*, Figs. S1 and S2 and Table S1, shows that glycolipids selectively increase the activation enthalpy of lateral proton diffusion while leaving the barrier for surface-to-bulk release largely unchanged.

## Discussion

Lipid-anchored sugars affected the Gibbs activation free-energy barrier Δ*G*^‡^ for proton release from the membrane surface into the bulk only marginally. Even at the highest glycolipid concentration investigated—corresponding to approximately one sugar moiety per two lipid molecules—Δ*G*^‡^ remained close to 30 kT (Table 1). Similar values were obtained for neutral, positively charged, negatively charged, and glycosylated membranes. Thus, the barrier opposing proton release from the membrane surface remains largely intact even in glycolipid-rich membranes. This finding implies that glycolipid-rich membranes, such as thylakoid membranes, are well suited to support localized proton coupling between membrane-embedded proton sources and sinks.

Temperature-dependent measurements (*SI Appendix*, Figs. S1 and S2) further indicate that this surface-to-bulk release barrier is dominated by entropic rather than enthalpic contributions, consistent with previous studies (26). In contrast, interfacial sugar moieties significantly increased the activation enthalpy for lateral proton diffusion, Δ*H*^‡^_d_. Unlike charged lipids, which affect Δ*H*^‡^_d_ only weakly, glycolipids increased Δ*H*^‡^_d_ beyond a few kT, i.e., to an energy scale exceeding that of a single hydrogen bond (*SI Appendix*, Table S1).

This increase in Δ*H*^‡^_d_ is accompanied by a pronounced reduction in lateral proton diffusivity. *D*_2_ (*SI Appendix*, Table S1) decreases with increasing glycolipid concentration and reaches less than one third of its value in neutral DOPC membranes at the highest sugar content. These observations support the conceptual framework outlined in the Introduction (Fig. 1*B*): membrane-anchored sugars slow lateral proton migration by increasing proton residence times at interfacial hydration sites rather than by promoting proton release into the bulk.

The stronger effect of DGDG compared with SQDG indicates that proton slowing is not governed primarily by the sulfonate group of SQDG. This conclusion is consistent with the modest influence of membrane charge observed for DOPG and DOTAP and with previous work showing that electrostatics have only a limited effect on lateral proton migration (26). Although electrostatic interactions may influence surface-to-bulk exchange, they do not significantly impede lateral proton propagation.

A more plausible explanation is that proton slowing arises from the spatial organization of hydroxyl groups at the membrane interface. DGDG contains two galactose rings with multiple hydroxyl groups distributed over an extended headgroup. Interactions between these hydroxyl groups and interfacial water are expected to create a heterogeneous interfacial environment with reduced orientational coherence of water dipoles, consistent with the reduced hydration repulsion and aggregation tendency of DGDG-rich membranes (48–52). In such an environment, migrating protons may interact more frequently with transient hydrogen-bond sites, increasing their residence time and thereby reducing lateral mobility.

In contrast, SQDG contains fewer hydroxyl groups and carries a negatively charged sulfonate group, which promotes a more strongly hydrated and electrostatically repulsive interface. These structural differences provide a plausible basis for the less pronounced changes in *D_2_* and Δ*H*^‡^_d_ observed for SQDG-containing membranes.

The presence of hydroxyl groups alone is insufficient to slow proton diffusion. For example, glycerol monooleate membranes, which also contain hydroxyl groups, do not show a comparable reduction in *D_2_* (16). In this case, the hydroxyl groups are confined to a small and flexible headgroup, and their interaction with interfacial water does not substantially disrupt the organization of the hydration layer. As in phospholipid membranes, rapid proton migration can therefore proceed predominantly through interfacial water beyond the tightly bound first hydration shell (29, 53), a mechanism that also applies at hydrophobic interfaces such as decane–water (20).

It is important to distinguish equilibrium proton populations from the nonequilibrium transport process measured here. Previous studies have identified multiple interfacial proton environments, including more strongly associated states near lipid headgroups and carbonyl groups (54, 55), which contribute to the equilibrium surface affinity of protons (15, 56). In our experiments, however, protons are generated locally at the membrane surface and their arrival at a distant site is monitored. The measured *D_2_* therefore reflects the mobile proton population responsible for long-range lateral transport (21, 26). Strongly bound protons are expected to contribute little, as their motion is coupled to the comparatively slow lipid matrix. This interpretation is consistent with simulations showing limited lateral displacement of surface-bound protons (54, 57, 58).

The relative populations of weakly and strongly bound interfacial protons may depend on pH and on local membrane structure, including defects that can alter proton binding environments. Equilibrium studies have indeed suggested pH-dependent regimes of proton dynamics (56), and analyses invoking defect-mediated trapping have emphasized the role of structural heterogeneities in modulating interfacial proton populations (59). These approaches, however, are based on equilibrium partitioning between proton states or assume quasi-equilibrium conditions at the interface.

In contrast, the present measurements probe long-range proton propagation under nonequilibrium conditions, where protons are locally introduced at the membrane surface and transport is dominated by the mobile interfacial proton population rather than by equilibrium distributions among bound states (21). Indeed, previous work has demonstrated that equilibrium-based models fail to quantitatively describe proton propagation under such conditions, whereas nonequilibrium descriptions capture both the kinetics and spatial characteristics of interfacial proton transport (21).

Consistent with this view, proton migration at the decane–water interface exhibits similar *D_2_* and Δ*G*^‡^ values despite the absence of lipids (20), and simulations indicate that even in the presence of trapping sites, the mobile proton population governs lateral transport (29).

We anticipate that lateral proton migration may be further inhibited at higher local glycolipid densities, such as in glycolipid-rich domains, where diffusion could become spatially heterogeneous. It will be of interest to determine whether such structuring correlates with the localization of proton pumps and sinks in biological membranes.

Finally, our ionophore-based approach releases protons directly at the membrane surface. Using the Ca^2+^/H^+^ exchanger A23187 avoids ambiguities associated with proton release into the bulk in microinjection or uncaging approaches (60, 61). Because Ca^2+^ diffusion remained unaffected, convection can be excluded. The agreement with previous measurements using caged compounds (26) demonstrates that both approaches yield consistent and robust values for interfacial proton transport.

Taken together, our results show that membrane charge has only a minor effect on proton transport, whereas glycolipid headgroups markedly reduce lateral proton mobility while leaving the surface-to-bulk release barrier essentially unchanged. These findings support a model in which membrane-anchored sugars tune long-range proton conduction without compromising proton retention (i.e., delayed surface-to-bulk release) at the membrane interface.

## Materials and Methods

Horizontal planar lipid bilayer membranes were formed from a lipid mixture consisting of 1,2-dioleoyl-sn-glycero-3-phosphocholine (DOPC, Sigma-Aldrich), 1,2-dioleoyl-sn-glycero-3-phospho-(1′-rac-glycerol) (DOPG, Sigma-Aldrich), 1,2-dioleoyl-3-trimethylammonium-propane (DOTAP, Sigma-Aldrich), digalactosyldiacylglycerol (DGDG, Avanti Polar Lipids), and sulfoquinovosyldiacylglycerol (SQDG, Avanti Polar Lipids), all dissolved in n-decane at a concentration of 20 mg/mL. The lipid solution was supplemented with 3% of the pH-sensitive fluorescent lipid dye fluorescein-1,2-dihexadecanoyl-sn-glycero-3-phospho-ethanolamine (fluorescein-DHPE, Thermo Fisher Scientific) and with the calcium ionophore A23187 (Sigma-Aldrich) at a final concentration of 5 mM.

Bilayer membranes were generated by spreading the lipid solution across a circular aperture (180 to 250 µm in diameter) in a Teflon septum separating two aqueous chambers. The cis chamber contained 50 mM sodium chloride (NaCl) and 0.1 mM N-cyclohexyl-3-aminopropanesulfonic acid (CAPSO), adjusted to pH 9. For experiments involving the calcium-sensitive dye Fluo-3 (Enzo Life Sciences), the dye was added directly to this buffer at a final concentration of 5 µM. The trans chamber contained 50 mM NaCl, 5 mM tris(hydroxymethyl)aminomethane (TRIS), and 0.015 mM ethylene glycol-bis(β-aminoethyl ether)-N,N,N′,N′-tetraacetic acid (EGTA), adjusted to pH 8.0.

A heat-pulled glass micropipette with a tip diameter of 1.5 to 2 µm, filled with 1 M calcium chloride (CaCl_2_), was used to deliver a localized calcium pulse near the membrane surface. Approximately 6 to 8 fL of solution was released. Calcium was subsequently transported across the membrane via A23187, coupled to countertransport of protons. The injected volume was determined by expelling the solution into n-decane and calculating the average volume of the resulting bubbles. A defined membrane patch at a specified distance from the calcium release site was illuminated at 488 nm, and the resulting fluorescence was detected using a photon-counting module (Picoquant, Berlin, Germany). The arrival of the proton wave at the detection site manifested as changes in fluorescence intensity, from which diffusion and release rate constants were derived.

## Supplementary Material

Appendix 01 (PDF)

## Data Availability

All study data are included in the article and/or *SI Appendix* and have been deposited in Zenodo at https://doi.org/10.5281/zenodo.20829855 (62).
