# Supplementary material for: Glycolipids slow interfacial proton migration while preserving surface proton retention
Source: Proc Natl Acad Sci U S A. 2026 Jul 8;123(28):e2537390123. doi: 10.1073/pnas.2537390123 (PMC13367777; doi:10.1073/pnas.2537390123)
Supplement: Supplementary file 1 — Appendix 01 (PDF) [file pnas.2537390123.sapp.pdf]

PNAS

Supporting Information for

Glycolipids slow interfacial proton migration while preserving surface proton retention.

Anna Maznichenko, Peter Pohl

Email: peter.pohl@jku.at

This PDF file includes:

Supporting text

Fig. S1

Fig. S2

Table S1

SI References

Lateral proton transport is selectively slowed by glycolipids due to an increased activation enthalpy, while the surface-to-bulk release barrier remains largely unchanged.

To assess the temperature dependence of proton diffusion, experiments on membranes containing high fractions of glycolipids were repeated at 16 °C and 26 °C using an external temperature control unit, following the procedure described in the main text. Each dataset (Fig. S1A and Fig. S2A) was fitted individually using the non-equilibrium diffusion model (Eq. 4 in the main text) to extract the lateral diffusion coefficient D_2 and the proton release rate constant k_{off}. Data at 19 °C were taken from the experiments described in the main text (Fig. 5 and Fig. 6). The amplitudes were normalized to 1, since only D_2 and k_{off} obtained from the fits were used for further analysis.

D_2 and k_{off} were subsequently used to determine activation enthalpies from their temperature dependence using Arrhenius relations:

$$k_{\mathrm{off}} = A_k \exp\left(-\frac{\Delta H^{\ddagger}{}_k}{k_{\mathrm{B}} T}\right) \tag{S1}$$

$$D_2 = A_D \exp\left(-\frac{\Delta H^{\ddagger}{}_d}{k_{\mathrm{B}} T}\right) \tag{S2}$$

where A_k and A_D are pre-exponential factors. Here, $\Delta H_k^{\ddagger}$ and $\Delta H_D^{\ddagger}$ denote the activation enthalpies associated with proton release from the membrane surface into the bulk (as described by k_{off}) and with lateral proton diffusion along the membrane (as described by D_2), respectively. This analysis yielded activation enthalpies $\Delta H^{\ddagger}{}_k$ = 8.2±0.3 kT and $\Delta H^{\ddagger}{}_d$ = 7.3±0.3 kT for membranes containing 25% SQDG and $\Delta H^{\ddagger}{}_k$ = 3.5±0.6 kT and $\Delta H^{\ddagger}{}_d$ = 11.0±1.4 kT for membranes containing 25% DGDG (Fig. S1B, Fig. S2B). $\Delta H^{\ddagger}{}_k$ values remain on the order of a few kT, comparable to the strength of a hydrogen bond at room temperature.

To separate enthalpic and entropic contributions to the activation barrier for proton release, the entropic term was estimated from the pre-exponential factors. Following the framework outlined in the main text and previous work (1, 2), the attempt frequency for proton transfer at the interface can be related to the diffusion prefactor A_D. This allows estimation of the activation entropy:

$$T\Delta S_k^{\ddagger} = k_B T \ \ln\left(\frac{A_k}{\nu_k}\right) \tag{S3}$$

where ν_k is the effective attempt frequency for interfacial proton transfer. The resulting values were $\Delta S_k^{\ddagger}$ =−23.6±0.4 k for SQDG-containing membranes and $\Delta S_k^{\ddagger}$=−30.9±1.6 k for DGDG-containing membranes. The attempt frequency for proton transfer can be related to the diffusion prefactor A_D via the Einstein relation for hopping diffusion:

$$\nu_k = \frac{4A_D}{l^2} \tag{S4}$$

where l = 2.8 Å is the characteristic O–O distance between neighboring water molecules.

Finally, the Gibbs activation free energy barrier for proton release was obtained using:

$$\Delta G_k^{\ddagger} = \Delta H_k^{\ddagger} - T\Delta S_k^{\ddagger} \tag{S5}$$

yielding $\Delta G_k^{\ddagger}$=31.8±0.5 kT for SQDG-containing membranes and $\Delta G_k^{\ddagger}$=34.4±1.7 kT for DGDG-containing membranes.

The thermodynamic parameters derived from this analysis are summarized in Table S1. While the activation enthalpy associated with proton release, $\Delta H^{\ddagger}{}_k$, remains small and varies only weakly with membrane composition, a pronounced increase in the activation enthalpy for lateral proton diffusion, $\Delta H^{\ddagger}{}_d$ is observed for membranes containing glycolipids compared to both neutral (DOPC) and charged (DOPG, DOTAP) membranes.

This increase in $\Delta H^{\ddagger}{}_d$ reflects stronger interactions of protons with interfacial sites, consistent with an increased residence time during proton hopping along the membrane. As a consequence, lateral proton mobility is reduced, as evidenced by the decreased diffusion coefficients D_2 (Table S1). These findings demonstrate that glycolipid headgroups selectively affect the energetics of lateral proton transport without significantly altering the barrier for surface-to-bulk release.

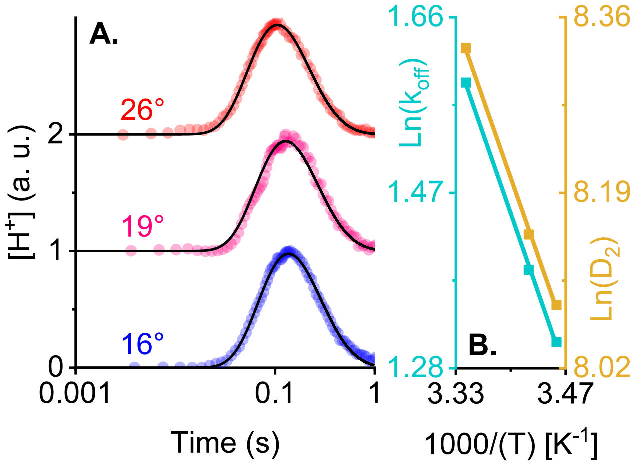

Figure S1. Temperature dependence of lateral proton migration containing 25% SQDG. A. Proton concentration, [H$^+$], measured at a remote detection site as a function of time after proton release for three temperatures. The distance between release and detection sites was 54 µm. Data were normalized to unity and vertically offset for clarity. Solid lines represent fits using the two-dimensional diffusion model (Eq. 4 in the main text). **B.** Arrhenius plots of the release rate constant k_{off} (turquoise) and the lateral diffusion coefficient D_2 (yellow). Linear fits yield activation enthalpies of $\Delta H^{‡}_{k}$ = 8.2±0.3 kT and $\Delta H^{‡}_{d}$ = 7.3±0.3 kT.

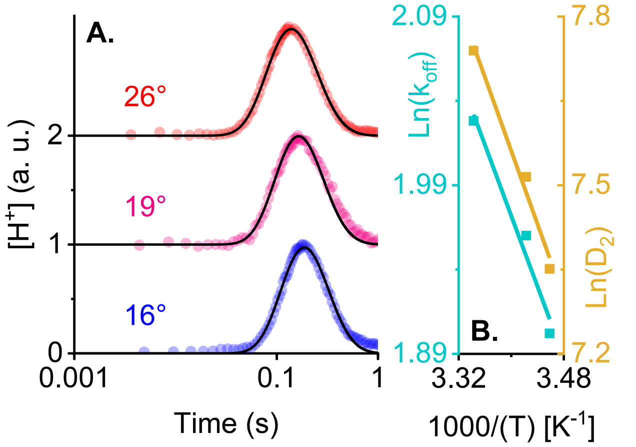

Figure S2. Temperature dependence of lateral proton migration in membranes containing 25% DGDG. A. Proton concentration [H$^+$] measured at a remote detection site as a function of time after proton release for three temperatures. The distance between release and detection sites was 54 µm. Data were normalized to unity and vertically offset for clarity. Solid lines represent fits using the two-dimensional diffusion model (Eq. 4 in the main text). **B.** Arrhenius plots of the release rate constant k_{off} (turquoise) and the lateral diffusion coefficient D_2 (yellow). Linear fits yield activation enthalpies of $\Delta H^{‡}_{k}$ = 3.5±0.6 kT and $\Delta H^{‡}_{d}$ = 11.0±1.4 kT.

Table S1. Thermodynamic parameters describing proton release and lateral diffusion at membrane interfaces. The table lists the surface proton diffusion coefficients D_2 (at 19 °C), the activation enthalpies for lateral diffusion $\Delta H^{\ddagger}_{d}$, the activation enthalpies for surface-to-bulk release $\Delta H^{\ddagger}_{k}$, the entropic contributions $T\Delta S^{\ddagger}_{k}$, and the corresponding Gibbs activation free-energy barriers ($\Delta G^{\ddagger}_{k}$) for proton release. Parameters were obtained from Arrhenius analysis of the temperature dependence of k_{off} and D_2 (Fig. S1, Fig. S2). Subscripts *k* and *d* refer to proton release into the bulk and lateral diffusion along the membrane, respectively. Values for DOPC are taken from (1), and those for DOPG and DOTAP/DOPC from (2). Lipid compositions are given in mol%; the remaining fraction consists of DOPC.

**Lipid**	**D_2 (µm²/s),**	**$\Delta H^{\ddagger}_{d}$ (kT)**	**$\Delta H^{\ddagger}_{k}$ (kT)**	**$T\Delta S^{\ddagger}_{k}$ (kT)**	**$\Delta G^{\ddagger}_{k}$ (kT)**
DOPC	5338 ± 64	5.9 ± 1.1	5.7 ± 0.7	-26.1	31.8
DOPG	4650 ± 39	4.5 ± 1.2	13.0 ± 3	-20.0 ± 3	33.0
DOTAP/ DOPC 50 %	5209 ± 35	6.6 ± 0.9	0 ± 3	-30.0 ± 3	30.0
SQDG 25%	3462 ± 25	7.3 ± 0.3	8.2 ± 0.3	-23.6±0.4	31.8±0.5
DGDG 25%	1835 ± 19	11.0 ± 1.4	3.5 ± 0.6	-30.9±1.6	34.4±1.7

Notably, the activation enthalpy for proton release, $\Delta H^{\ddagger}_{\text{k}}$, is higher for SQDG-containing membranes than for DGDG-containing membranes. This difference is consistent with the presence of negatively charged headgroups in SQDG, which electrostatically stabilize protons at the membrane interface. In this framework, $\Delta H^{\ddagger}_{\text{k}}$ can be understood as the sum of an intrinsic contribution and an electrostatic term arising from the membrane surface potential, such that $\Delta H^{\ddagger}_{\text{k}} = \Delta H^{\ddagger}_{\text{r}} - e\psi_0$, where $e\psi_0$ represents the energy of the interfacial electric field acting on the proton. The larger $\Delta H^{\ddagger}_{\text{k}}$ observed for SQDG membranes is therefore consistent with an additional energetic penalty required for proton release against this attractive electrostatic potential. At the same time, these electrostatic contributions primarily affect the enthalpic component of the barrier, while compensatory changes in entropy ensure that the overall free-energy barrier $\Delta G^{\ddagger}_{\text{k}}$ remains largely unchanged.

The Gibbs activation energies $\Delta G^{\ddagger}_{\text{k}}$ obtained from temperature-dependent analysis are in reasonable agreement with $\Delta G^{\ddagger}$ calculated from k_{off} using transition state theory, although small deviations are observed. These differences likely arise because the Arrhenius analysis separates enthalpic and entropic contributions under the assumption of temperature-independent prefactors, whereas the transition state expression assumes a universal attempt frequency. In addition, experimental uncertainties in k_{off} and the limited temperature range can further contribute to deviations. Importantly, both approaches consistently indicate that $\Delta G^{\ddagger}_{\text{k}} \approx \Delta G^{\ddagger}$ remains largely unaffected by membrane composition.

References

1. E. Weichselbaum *et al.*, Origin of proton affinity to membrane/water interfaces. *Sci. Rep.* **7**, 4553 (2017).
2. E. Weichselbaum, T. Galimzyanov, O. V. Batishchev, S. A. Akimov, P. Pohl, Proton Migration on Top of Charged Membranes. *Biomolecules* **13**, 352 (2023).
